# Standardization and validation of a high-efficiency liquid chromatography with a diode-array detector (HPLC-DAD) for voriconazole blood level determination

**DOI:** 10.7705/biomedica.6959

**Published:** 2024-03-31

**Authors:** Juan D. Zapata, Diego H. Cáceres, Luz E. Cano, Catalina de Bedout, Sinar D. Granada, Tonny W. Naranjo

**Affiliations:** 1 Unidad de Micología Médica y Experimental, Corporación para Investigaciones Biológicas, Medellín, Colombia Corporación para Investiga. Biológicas Unidad de Micología Médica y Experimental Corporación para Investigaciones Biológicas Medellín Colombia; 2 Unidad de Investigación Clínica, Corporación para Investigaciones Biológicas, Medellín, Colombia Corporación para Investiga. Biológicas Unidad de Investigación Clínica Corporación para Investigaciones Biológicas Medellín Colombia; 3 Escuela de Microbiología, Universidad de Antioquia, Medellín, Colombia Universidad de Antioquia Escuela de Microbiología Universidad de Antioquia Medellín Colombia; 4 Unidad de Fitosanidad y Control Biológico, Corporación para Investigaciones Biológicas, Medellín, Colombia Corporación para Investiga. Biológicas Unidad de Fitosanidad y Control Biológico Corporación para Investigaciones Biológicas Medellín Colombia; 5 Escuela de Ciencias de la Salud, Universidad Pontificia Bolivariana, Medellín, Colombia Pontificia Universidad Bolivariana Escuela de Ciencias de la Salud Universidad Pontificia Bolivariana Medellín Colombia; 6 Unidad de Biología de Sistemas, Escuela de Ciencias de la Salud, Universidad Pontificia Bolivariana, Medellín, Colombia Pontificia Universidad Bolivariana Unidad de Biología de Sistemas Escuela de Ciencias de la Salud Universidad Pontificia Bolivariana Medellín Colombia; * These authors contributed equally on this project.

**Keywords:** Antifungal agents, voriconazole, chromatography, high pressure liquid, drug monitoring, antifúngicos, voriconazol, cromatografía líquida de alta presión, monitoreo de medicamentos.

## Abstract

**Introduction.:**

A specialized service for antifungal blood level determination is not available in Colombia. This service is essential for the proper follow-up of antifungal therapies.

**Objective.:**

To standardize and validate a simple, sensitive, and specific protocol based on high-performance liquid chromatography with a diode array detector for voriconazole blood level quantification.

**Materials and methods.:**

We used an Agilent HPLC™ series-1200 equipment with a UV- diode array detector with an analytical column Eclipse XDB-C18 and pre-column Eclipse- XDB-C18 (Agilent). We used voriconazole as the primary control and posaconazole as an internal control. We performed the validation following the Food and Drug Administration (FDA) recommendations.

**Results.:**

The best chromatographic conditions were: Column temperature of 25°C, UV variable wavelength detection at 256 nm for voriconazole and 261 nm for posaconazole (internal standard); 50 μl of injection volume, 0,8 ml/min volume flow, 10 minutes of run time, and mobile phase of acetonitrile:water (60:40). Finally, retention times were 3.13 for voriconazole and 5.16 minutes for posaconazole. Quantification range varied from 0.125 μg/ml to 16 μg/ml.

**Conclusion.:**

The selectivity and chromatographic purity of the obtained signal, the detection limits, and the standardized quantification make this method an excellent tool for the therapeutic monitoring of patients treated with voriconazole.

## Resumen

**Introducción.:**

Hasta la fecha, Colombia no cuenta con un servicio especializado de medición de niveles séricos de antifúngicos, procedimiento esencial para el adecuado seguimiento del tratamiento de infecciones fúngicas invasoras.

**Objetivo.:**

Estandarizar y validar un protocolo -simple, sensible y específico- basado en la aplicación de cromatografía líquida de alta eficiencia acoplada con un detector de arreglo de diodos para la cuantificación de los niveles séricos de voriconazol.

**Materiales y métodos.:**

Se usó un equipo HPLC-Agilent^TM^, serie-1200, con un detector UVDAD, una columna analítica Eclipse-XDB-C18 y una pre-columna Eclipse-XDB-C18, ambas de la marca Agilent. Como control primario se utilizó voriconazol y como control interno, posaconazol. La validación se hizo cumpliendo todos los criterios de aceptación recomendados por la *Food and Drug Administration* (FDA).

**Resultados.:**

Las mejores condiciones cromatográficas se obtuvieron con los siguientes parámetros: temperatura de la columna de 25 °C, detección UV-VWD de 261 nm, volumen de inyección de 50 μl, flujo de 0,8 ml/minuto y un tiempo de corrido de 10 minutos. La fase móvil usada fue acetonitrilo:agua (60:40) y los tiempos finales de retención fueron de 3,13 para voriconazol y de 5,16 minutos para posaconazol. El rango de cuantificación fue desde 0,125 μg/ml hasta 16 μg/ml.

**Conclusiones.:**

La selectividad y la pureza de la señal cromatográfica, así como los límites de detección y cuantificación estandarizados hacen de esta metodología una excelente herramienta para el seguimiento terapéutico de pacientes tratados con voriconazol o en profilaxis con este fármaco.

Voriconazole is an azole drug approved by the Food and Drug Administration (FDA) for human use in 2002 ^1^. This antifungal is indicated to treat invasive fungal infections caused by *Aspergillus* spp., *Candida* spp., *Fusarium* spp., and *Scedosporium* spp. Additionally, it is an alternative drug for prophylaxis in patients at risk of developing an invasive fungal infection, such as bone marrow transplant recipients, those with hematological malignancies, or prolonged neutropenia due to chemotherapy ^2^^,^^3^. The voriconazole mechanism of action is similar to that of other azole drugs: inhibiting the synthesis of ergosterol by binding to the lanosterol-14a-demethylase enzyme present in most fungi except the genus *Pneumocystis* and *Phytium*^4^^).^

Unlike other azoles, voriconazole is mostly unmetabolized, and the body eliminates it without any changes. The low metabolism predominantly occurs through the UDPG-transferase pathway and not by the enzymes of the cytochrome P450 system, although it does have the ability to inhibit the CP3A4 system. Therefore, in a smaller proportion to other azoles, it presents problems of drug interactions ^1^^-^^3^. As for all antimicrobial medications, voriconazole must reach effective blood levels to prevent treatment failures. Since this antifungal presents a high pharmacokinetic variability between patients, its prescription is accompanied by several recommendations to increase absorption efficiency and preventive use of drugs that can affect voriconazole blood concentrations.

Monitoring antifungal blood levels during therapy is recommended ^2^^,^^5^, but despite the recommendation, measurement of voriconazole blood levels is limited in Colombia. For all the above, the objective of this study was to standardize and validate a bio-analytical method using high-efficiency liquid chromatography (HPLC) for voriconazole determination and quantification in human serum. The *Instituto de Salud Carlos III* previously developed the method validated in this study ^6^. This study followed the criteria and parameters recommended by the FDA of concerning analytical methodologies validation ^7^.

## Materials and methods

### Standards and reagents

We used voriconazole with 100% purity, lot number 10264013, donated by Pfizer. As an internal control, posaconazole (SCH56592) with 100% purity, lot number IRQ-PAZ-10-X-103, donated by Merck Sharp and Dohme Corporation. We also used dimethyl sulfoxide (DMSO), Carlos Erba, reference number 445103; HPLC acetonitrile, Merck, reference number 1.00030.5000; type 1 MilliQ Direct 16 water, Millipore; and Agilent Captiva cellulose filters of 0.45 pm pore from Agilent technologies, reference number 5190-5109.

### HPLC equipment and chromatographic conditions

We used an Agilent 1200 series HPLC equipment with a UVDAD detector, an Eclipse XDB C18 analytical column (4.6 x 150 mm, 5 μm), and an Eclipse- XDB-C18 pre-column (Agilent Technologies, Germany). During the analytical method standardization, we conducted different tests to obtain the best conditions of mobile phase, temperature, wavelength, running volume, flow, run time, and detection times to allow accurate identification of voriconazole.

### Preparation of standard solutions

For the primary standard preparation, 3.2 mg of voriconazole were diluted in 2 ml of DMSO, stirred for 2 minutes in a vortex, and then subjected to ultrasound for 5 minutes, obtaining a stock solution with a 1.6 mg/ml concentration. We prepared two standard curves from this stock solution: one using acetonitrile:water as a diluent (60:40) and another using human serum. We took 10 μl from the stock solution and set a 1:100 dilution, constituting the highest concentration point (16 μg/ml) to perform the calibration curve. The other points were prepared by making base two serial dilutions until reaching the lowest concentration tested in the curve (0.125 μg/ml). In the case of the internal standard, we diluted 2.0 mg of posaconazole in 1 ml of DMSO, stirred in a vortex for 2 minutes, then subjected to ultrasound for 5 minutes. We prepared a dilution 1:10 from the previous solution using DMSO to obtain a 200 μg/ml stock solution of the internal standard.

### Preparation specimens

For quality control, we prepared three human sera by doping it with voriconazole and obtaining the same number of serum with different concentrations: a low concentration of 0.25 μg/ml, a mild concentration of 8.0 μg/ml, and a high concentration of 16.0 μg/ml. Once we obtained the doped samples, the extraction process consisted of taking 150 μl of each specimen and adding 3 μl of the internal standard of posaconazole stock solution. We added 147 μl of cold acetonitrile to the previous mixture and mixed it by vortex for 30 seconds, followed by centrifugation at 13,800 rpm for 15 minutes to induce precipitation. We filtered the obtained supernatant through a 0.45 pm membrane and injected 50 μl of this filter supernatant into the HPLC equipment.

### Validation of the analytical method

We did the analytical method validation following the parameters recommended by the FDA. These recommendations include the evaluation of the selectivity, the calibration curve, the percentage of recovery, specimen stability, and the calculation of accuracy, precision, sensitivity, and reproducibility ^7^.

### Ethical statements

This study did not require ethical approval.

## Results

### Standardization of the HPLC method for voriconazole

The best chromatographic conditions to determine voriconazole blood levels were: column temperature of 25°C, UV-VWD detection at 256 nm for voriconazole and 261 nm for posaconazole, injection volume of 50 μl, flow of 0.8 ml/minute, running time of 10 minutes, and mobile phase of acetonitrile:water (60:40). Retention times were 3.13 minutes for voriconazole and 5.16 minutes for posaconazole (internal standard) (table 1).

**Table 1:** Chromatographic conditions for the determination of voriconazole in sera samples

Parameter	Characteristics
Method	Isocratic 60:40 (acetonitrile:water)
Flow	0,8 ml/min
Injection volume	50 μl
Detection (λ)	UV: 256 nm for voriconazole, and 261 nm for posaconazole (internal standard)
Column	C18, 150 x 4,6 mm, particle size: 5 μm (Agilent Technologies, ref. Eclipse plus C18)
Precolumn	C18, 20 x 4,6 mm, particle size: 5 μm (Agilent Technologies)
Column temperature	25°C
Internal standard	Posaconazole
Units of concentration	μg/ml
Retention time (min)	Voriconazole: 3,130
	Posaconazole: 5,160
Running time (min)	10,0

### Method validation according to the FDA parameters

*Selectivity:* We demonstrated the selectivity of the chromatographic analytical method by the lack of peaks of possible endogenous compounds or interferents. Results were the same on chromatograms of six blank specimens. The internal standard of posaconazole worked every time.

**Figure 1.**
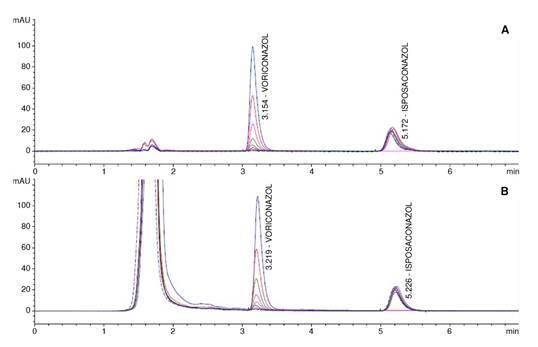
Chromatograms showing the detected signal (Y axis) versus retention times (X axis) for voriconazole and its internalstandard posaconazole. A: Analytes diluted in diluent solution (acetonitrile:water; 60:40). B: Analytes diluted in human sera.

*Calibration curves:* We tested two calibration curves, one prepared using acetonitrile:water (60:40) and another with human sera. Calibration curves were subjected to the extraction process and then analyzed. We assessed each of the curves by triplicate with eight concentrations of voriconazole distributed over the range of 0.125 μg/ml, 0.25 μg/ml, 0.5 μg/ml, 1.0 μg/ml, 2.0 μg/ml, 4.0 μg/ml, 8.0 μg/ml, and 16.0 μg/ml. The coefficient of determination R^2^ was always greater than 0.99 for all curves (figure 1). The relative percentage error of each point of the two calibration curves was less than 15%, meeting the parameter recommended by the FDA.

*Recovery percentage:* This parameter was determined in three specimens with different voriconazole concentrations: 0.25 μg/ml (low), 8.0 μg/ml (mild), and 16.0 μg/ml (high). We calculated the recovery percentage by comparing the responses (in terms of area) obtained for the voriconazole extracted from the biological matrix (sera) and the one obtained from the same concentrations of the calibration curve standard solutions. Although this parameter is irrelevant for validation, we found a reproducible percentage. The recovery percentage for voriconazole was 98.7%, with an overall coefficient of variation of 6.7%.

*Accuracy and precision:* These parameters were evaluated intra and inter-assay using data obtained from experiments on three different days. We prepared three serum specimens with diverse concentrations of voriconazole: 0.25 μg/ml (low), 8.0 μg/ml (mild), and 16.0 μg/ml (high). The accuracy values of relative percentage error and calibration curves were below 15%, meeting the parameters recommended by the FDA.

*Sensitivity:* The limits of detection and quantification were calculated using the method based on extrapolation of the calibration line to zero concentration. These analyses were done in triplicate, including a three-point serum calibration curve with a low concentration close to the limit of quantification of the method (0.125 μg/ml). With this curve, the background signal and the standard deviation were calculated. The limits of detection and quantification for the bioanalytical assay were 0.03 μg/ml and 0.125 μg/ml, respectively.

*Specimen stability:* Specimens were stored in the automatic injector for 24 hours, 48 hours, and five days at room temperature (23°C) and then frozen at -20°C. Concentrations did not present more than 15% of variation.

## Discussion 

Following the parameters recommended by the FDA ^7^, we standardized and validated a method based on HPLC capable of detecting and quantifying voriconazole blood levels. This method is simple, economical, rapid, and highly accurate, with a wide and reliable quantification range varying from 0.125 μg/ml to 16 μg/ml of voriconazole.

The selectivity and purity of the obtained chromatographic signal and the standardized detection and quantification limits make this methodology an excellent tool for the therapeutic monitoring of patients under treatment with voriconazole. The specimen pretreatment by protein precipitation with acetonitrile is simple, rapid, and highly reproducible. The use of a mobile phase, which does not require buffer solution preparation, significantly simplifies the testing, and reduces the time of work and costs. Stability analysis evidenced that voriconazole is stable on specimens, facilitating transport and handling from the collection site to the testing laboratory. Similar to other studies validating the HPLC method for the voriconazole blood level determination, we found this approach was robust, simple, accurate, and precise ^8^^-^^10^.

With the marked incidence increase of invasive fungal infections, the availability of sensitive and specific laboratory tests for quantification of voriconazole blood levels will help medical professionals make decisions for the welfare of patients. Drug monitoring would directly benefit the patient by reducing the risk of complications during treatment. Finally, most laboratories with medium capacity could implement this method.
